# Prevalence of Congenital Anomaly and Its Relationship with Maternal Education and Age According to Local Development in the Extreme South of Brazil

**DOI:** 10.3390/ijerph19138079

**Published:** 2022-07-01

**Authors:** Carolina Ribeiro Anele, Marcelo Zubaran Goldani, Lavínia Schüler-Faccini, Clécio Homrich da Silva

**Affiliations:** 1 Postgraduate Program in Child and Adolescent Health, Faculdade de Medicina, Universidade Federal do Rio Grande do Sul (UFRGS), Rua Ramiro Barcelos, 2400, Porto Alegre 90035-003, RS, Brazil; carolinaanele@gmail.com (C.R.A.); mgoldani@hcpa.edu.br (M.Z.G.); lavinia.faccini@ufrgs.br (L.S.-F.); 2 Pediatrics and Primary Health Care Service, Hospital de Clínicas de Porto Alegre (HCPA), Rua Ramiro Barcelos, 2350, Porto Alegre 90620-110, RS, Brazil; 3 Department of Pediatrics, Faculdade de Medicina, Universidade Federal do Rio Grande do Sul (UFRGS), Rua Ramiro Barcelos, 2400, Porto Alegre 90035-003, RS, Brazil; 4 Department of Genetics, Universidade Federal do Rio Grande do Sul (UFRGS), Avenida Bento Gonçalves, 9500, Porto Alegre 91501-970, RS, Brazil; 5 Instituto Nacional de Genética Médica Populacional (INAGEMP), Medical Genetics Service, Hospital de Clínicas de Porto Alegre, Rua Ramiro Barcelos, 2350, Porto Alegre 90035-003, RS, Brazil

**Keywords:** educational status, maternal age, congenital abnormalities, fetal diseases, vital statistics, health inequities, social conditions

## Abstract

Congenital anomalies (CA) contribute to disabilities and health conditions throughout life. Furthermore, they can cause emotional distress to the mothers and children, who may also experience limitations in individual and social development. This study investigated the prevalence of CA and the relationship with maternal education and age according to local development in the extreme south of Brazil. This is a retrospective observational study with birth data from the Live Birth Information System from 2000 to 2017. The association between age and maternal education with the presence of CA was verified using multiple Poisson regression for robust variances in models adjusted for those variables with a preliminary significant association. A total of 5131 (1.5%) had some CA identified at birth between 2000 and 2017. Only advanced age (≥36 years) was associated with CA regardless of macro-region development (*p* ≤ 0.001). The highest risk was observed in regions with medium development (RR = 1.60; 95% CI 1.30–1.97). Maternal education (<8 years of study) was associated with CA only in mothers from macro-regions with very high development (RR = 1.27; 95% CI 1.03–1.54). These analyses confirmed that women of advanced age are at greater risk of having children with a CA regardless of maternal education and local development, but social characteristics can also have an influence, as regions with higher development had lower prevalence of CA.

## 1. Introduction

Congenital anomalies (CA), also known as birth defects, congenital disorders, or congenital malformations, are defined as functional or structural alterations, many of which have a prenatal origin, and can be identified in this period, in the perinatal period, in the neonatal period, or even years after birth [1]. Structural CA can be classified into minor and major anomalies. Minor CA are those that do not pose health problems in the neonatal period or have minimal implications. Major CA, although less prevalent, significantly affect the health of the newborn and the future life of the individual with the need for medical interventions in most cases [1,2].

Approximately 50% of all birth defects have no defined cause; however, some genetic conditions, environmental agents, and infectious agents are known risk factors that can interfere with fetal development before, during, or after conception [3]. Many of the known causes can be prevented with vaccination and proper prenatal care, such as guidance on the use of safe medications during pregnancy [3,4,5]. Heart and neural tube defects and Down syndrome represent the most common severe CA [1]. In addition, the increased prevalence of gastroschisis, one of the abdominal wall defects, has been consistently documented in the literature [6,7].

It is estimated that about 7.9 million (6%) newborns worldwide have some congenital defect; this number is perhaps higher due to the difficulty in identifying cases in stillbirths or in interrupted pregnancies [1]. As most data are based on hospital records and therefore only relate to the first few days of the newborn’s life, prevalence estimates are limited to the neonatal period [8]. In Brazil, about 24,000 newborns are registered annually with some type of CA, and between 2010 and 2019, according to the latest epidemiological bulletin, the most prevalent among the eight priority groups were: limb defects (24.4 cases per 10,000 live births) followed by congenital heart disease (8.4/10,000 live births), oral clefts (6.1/10,000 live births), and genital organ defects (4.6/10,000 live births) [9].

In contrast to high-income countries, many of those in developing countries, such as Brazil, often have risk factors for pregnancy and some CA [10,11]. Teenage pregnancy, although it has decreased in recent years, is still a public health problem in the country. According to a population-based study that analyzed all Brazilian states, adolescent pregnancy rates vary from 39% to 75% among the total number of live births according to each geographical region of Brazil [12]. In this same perspective, a low educational level, seems to be associated with worse health care choices and therefore reflects worse social conditions of a mother, which are strongly related to harm to the child [13,14]. Data from the Brazilian Institute of Geography and Statistics (IBGE) show that, in 2019, the proportion of people aged 25 years and older who have completed compulsory basic education (finished high school) was 48.8%, and the illiteracy rate among women aged 15 years and older was 6.3%. Added to this, the country presents a high disparity between regions and states, which can be observed even within the same municipality, where some women are exposed to different risk scenarios such as lack of basic sanitation, adequate supplies, and limited access to health services [12,15].

CA represent one of the main causes of the total burden of disease and are associated with infant mortality, representing a significant portion of neonatal deaths. Data from the World Health Organization (WHO) for the period 2000 to 2016 show that each year, about 295,000 children die in the first 28 days after birth due to CA. In Brazil, CA are the second leading cause of death in children under five years of age [16]. These impacts are exacerbated in low- and middle-income countries, as these conditions most often present multiple health needs, demanding long-term specialized services that, in addition to increasing the expense of health systems, may be scarce in more precarious regions [10,17,18,19].

Given the discrepancy in existing living and health conditions among pregnant women, the present study investigated the prevalence of CA and the relationship to maternal education and age according to local development in a state capital in the far south of Brazil. Unlike most other studies that compare differences between countries, cities, and/or hospital type, this study analyzed the population of live births in the same city in a time series comparing regions according to the Municipal Human Development Index (MHDI).

## 2. Materials and Methods

### 2.1. Study Population and Data Collection

A retrospective observational study that included all live births from 2000 to 2017 was conducted in Porto Alegre, the capital city of Rio Grande do Sul, located in the extreme south of Brazil. The municipality has a population of 1,409,351 inhabitants distributed in an area of 471.85 km² divided into 17 macro-regions, each with a grouping of neighborhoods of similar characteristics [20]. The MHDI is very high (0.805), ranking seventh among Brazilian capitals [21]. Although the MHDI can be considered very high, the municipality has a Gini coefficient = 0.6029, which corresponds to one of the cities in the Southern Region with the highest inequality in income distribution [20].

The study used the annual databases of the Live Births Information System (SINASC) provided by the Vigilance Team of Vital Events, Noncommunicable Diseases and Conditions of the General Coordination of Health Surveillance of the Municipal Health Department of Porto Alegre after ethical approval. SINASC, supplied with information from the Declaration of Live Births (DN), is the system used in Brazil for epidemiological surveillance of newborns, providing knowledge of the conditions of prenatal care and childbirth. It was established in 1990 by the Ministry of Health and, as of 1998, the SINASC reached complete coverage in all states and municipalities of the country [22]. Newborns weighing less than 500 g and children of mothers not residing in the municipality of Porto Alegre were excluded from the study, due to the impossibility of assessing the development index of the region of domicile of other cities. The DN is a single document for each child, filled in shortly after birth by the care team, with maternal data, prenatal care data, and vital data on the newborn.

The presence of some CA detectable at birth according to the DN record was considered a dependent variable. The independent variables used were the home address and other maternal and perinatal variables.

The CA registered at SINASC are those diagnosed prenatally or at birth, so most of the CA identified at birth are structural. In the city of the study, almost all births took place in large hospitals, where the presence of the obstetrician and pediatrician was routine in the delivery room. There, the pediatrician identifies the CA through clinical examination, or prenatal diagnosis history, and even in the first hours of birth, the DN is filled out and the CA identification is recorded, if applicable.

The address of the mother’s home was used to verify the local development index by identifying the macro-region of each mother’s residence and then classifying it according to the corresponding value of the MHDI provided by the Atlas of Human Development in Brazil online platform [21]. The MHDI values were previously calculated nationwide by the United Nations Development Program (UNDP), the Institute for Applied Economic Research (IPEA), and the João Pinheiro Foundation based on the 2010 Demographic Census of the Brazilian Institute of Geography and Statistics (IBGE). Its classification is very low (0–0.499), low (0.5–0.599), medium (0.6–0.699), high (0.7–0.799), and very high (0.8–1.0). However, in Porto Alegre no macro-region has the MHDI classified as very low or low.

Maternal age was classified into three age group categories: ≤19 years—adolescence according to the WHO [23]; 20–35 years, and ≥36 years, considered advanced age at risk for conception according to the literature [5,7,24]. The other maternal variables used were: education in years of completed schooling (<8; 8–11; ≥12 years); marital status (married/stable union, single/separated/widowed); number of previous living children; number of previous dead children (none; ≥1); number of prenatal visits (None; 1–3; 4–6; ≥7); parity (primiparous; multiparous) according to the number of previous living children and number of previous dead children; gestation (single; multiple).

The perinatal variables used were: gestational age in weeks (<27, 28–31, 32–36, ≥37); hospital of birth (public, private, mixed); type of delivery (vaginal, cesarean); newborn weight (grams) for subsequent low birth weight classification (<2500 g); Apgar Index at the 5th minute of life (≥7, <7), and sex (female, male).

### 2.2. Data Analysis

A descriptive statistical analysis of risk factors was performed, presenting their absolute and relative frequencies according to the classification of local development of the maternal home. The prevalence of CA cases over the period (2000 to 2017) was evaluated by the Poisson regression model with robust variances. The quadratic term was used, as this model had a better fit than the one that considers linearity by Bayesian criterion [25]. The association between maternal age and education with the presence of CA was verified by means of multiple Poisson regression for robust variances in three models adjusted for those variables that showed significant association (*p* < 0.001) in the preliminary bivariate analysis by simple Poisson regression. Multiple comparisons were performed with Bonferroni correction. Statistical analysis was performed using IBM SPSS Statistics version 18.0 and R-studio version 4.1 software.

### 2.3. Ethical Aspects

We used the Statement of Commitment for the Use of Secondary Data, and the study was ethically approved by the Research Ethics Committees of the Hospital de Clínicas de Porto Alegre and the Secretaria Municipal de Saúde de Porto Alegre, under protocol numbers 2,940,235 (7 February 2019) and 3,153,671 (19 February 2019), respectively.

## 3. Results

**Figure 1:**
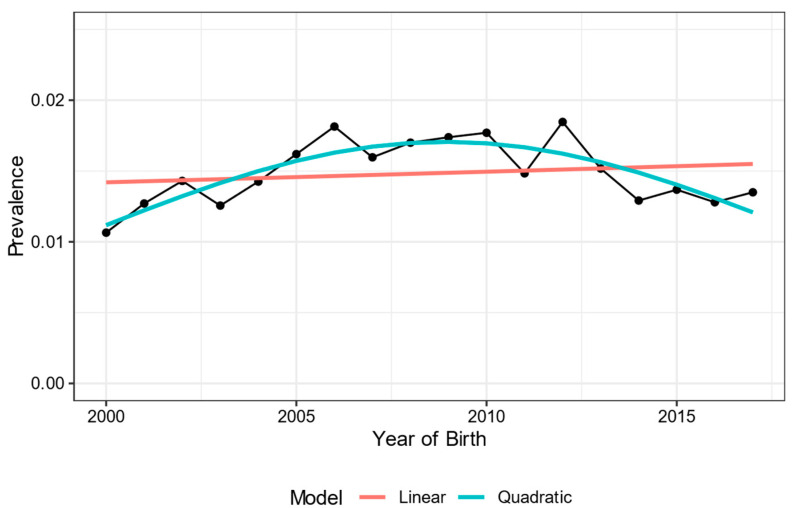
Prevalence of congenital anomaly per year among newborns in the city of Porto Alegre, Rio Grande do Sul, from 2000 to 2017 and model adjust (Bayesian criterion). Numerator: total number of newborns identified with CA. Denominator: total live births in the same year.

**Figure 2:**
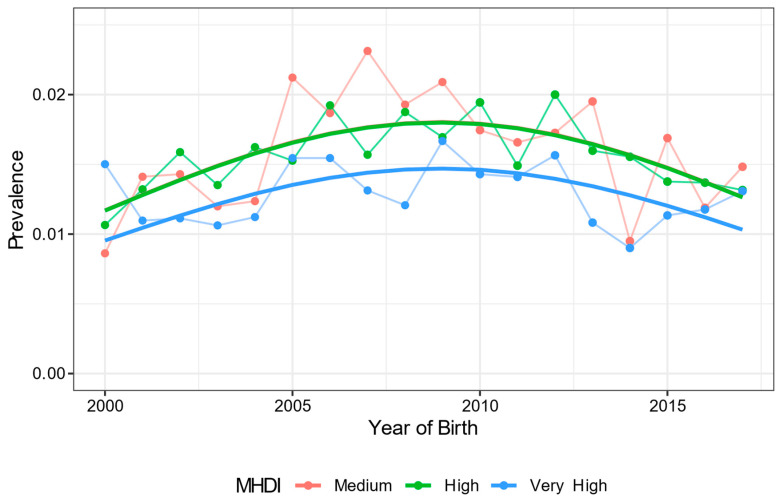
Prevalence of congenital anomaly per year among newborns according to the local development (MHDI classification) in the city of Porto Alegre, Rio Grande do Sul, from 2000 to 2017. Numerator: total number of newborns identified with CA. Denominator: total live births in the same year.

Of the 347,494 newborns included in the study, a total of 5131 (1.5%) had some CA identified at birth between 2000 and 2017. The prevalence of these CA showed variation in the analyzed period with some peaks of increase, but with a reduction from the year 2010 (Figure 1). There was no statistically significant difference in the prevalence of newborns with CA between regions with medium and those with high development (RR, 1.00 95% CI 0.91–1.10). However, the relative risk of CA was 1.23 (95% CI 1.10–1.37) and 1.22 (95% CI 1.23–1.33), respectively, in medium- and high-development regions compared to regions with very high development, which had a statistically lower frequency (*p* < 0.001) (Figure 2).

The proportion of CA was higher in women without a partner (single/separated or widowed), with less than eight years of education, in newborns from public hospitals, with low birth weight, lower gestational age, with Apgar score (5th minute) lower than seven, and male, regardless of the development of the maternal place of residence. The highest proportion of CA occurred in women aged ≥36 years in the medium (2.3%—*n* = 122) and high development (2.0%—*n* = 460) regions. Of mothers with less than eight years of education, the highest proportion of CA occurred in those who resided in very high development regions (2.0%—*n* = 276). A detailed statistical description of the other variables is presented in Table 1.

Regarding age, although the proportions of CA of mothers aged 10–19 years were high, after multiple analysis of each model adjusted for the other variables, only advanced age (≥36 years) showed an association with CA independent of macro-region development (*p* ≤ 0.001). Maternal education of less than eight years of schooling was associated with CA only in mothers from macro-regions with very high development with 1.27 higher risk (95% CI 1.03–1.54) (Table 2).

## 4. Discussion

In the present study, we observed that CA prevalence varied according to the hypothetical socioeconomic level of the mother through maternal home development, where regions with very high development showed statistically lower frequencies compared to the other regions. In addition, they also show lower frequencies of risk characteristics, such as teenage pregnancy (9.1%), lower educational level (15.5%), and lower number of prenatal visits. Advanced age (≥36 years) showed an association with CA in all regions, regardless of the local development classification, whereas low education (<8 years) showed an association with CA only in mothers residing in regions with very high development.

Analyses conducted in different locations around the world have shown variation in CA prevalence, where some CA show stability or a downward trend, and others have shown increases [9,26,27]. Although there are a predominant number of studies in the literature that point to the increasing prevalence of cases of gastroschisis, severe congenital heart disease, and Down syndrome [7,9,27,28], the variation in types of CA differ for each country or region, which reflects the common risk factors for each location. In a study with data from the European Congenital Anomalies Surveillance (EUROCAT) for the period from 1980 to 2012, Morris et al. [26] identified twelve subgroups of CA with an increasing trend, among them congenital heart defects, and other subgroups with a decrease, such as microcephaly, patent ductus arteriosus, congenital hydronephrosis, and limb reduction. Comparatively, in Brazil the anomalies with the largest percentage increases in the period from 2010 to 2019 were congenital heart defects, but also microcephaly, diverging from the trends shown elsewhere. The country showed a dramatic increase in microcephaly in the period from 2015 to 2016, as a consequence of the epidemic of Zika virus infections, which in turn is related to birth defects in the developing fetus, including microcephaly [9].

Unlike other countries, in Brazil, termination of pregnancy is not allowed, and the diagnosis of any CA is not a reason to perform the act. According to the Penal Code, abortion is considered a crime in Brazil, except in situations that put the woman’s life at risk, pregnancies resulting from rape, and if the fetus is anencephalic. This last condition is so rare that it would not occur in the prevalence observed in this study.

In Brazil, there were advances and setbacks in CA prevention during the analyzed period, which may have influenced the variation in CA prevalence. In 2004, the fortification with folic acid of wheat and corn flours, frequently consumed in the country, was decreed [29]. Likewise, since the early 2000s, the country has been implementing care protocols with guidelines on preconception approach, vaccination updates for women, and active search for pregnant women for prenatal care. The implementation of the Stork Network, launched by the federal government in 2011, promoted the expansion of the early identification and treatment of HIV and syphilis in pregnant women [30]. Particularly in Porto Alegre, the rates of vertical transmission between 2009 and 2010 were still around 6% of the total number of live births to infected mothers [20]. Currently, Brazil routinely performs serology for HIV and syphilis in the first and third trimester of pregnancy and at delivery. These measures have promoted improvements in maternal and child health, with a reduction in the prevalence of some CA. However, different exposure agents harmful to health have increased in recent years. In Porto Alegre, there has been an important increase in the use of illicit drugs in the last twenty years, mainly due to crack use, including an extensive number of women of childbearing age and in the gestational period [31]. Data from the National Survey on crack use in Brazil show that approximately 40% (95% CI 34.18–44.14) of users were in a street situation at the time of the survey; among women, the mean age was 29.60 years (95% CI 28.38–30.81), and 13% (95% CI 9.48–18.45) answered that they were pregnant at the time of the interview [32].

Two systematic reviews revealed that middle- and low-income countries usually have a lower prevalence of CA due to underreporting, contrary to the evidence on some risk factors present in these populations, such as poor diet and lower prenatal coverage. Data from these two studies show that in addition to underdeveloped countries presenting a fragility in the identification of birth defects, it is also common to have a high number of births that happen outside hospitals or health systems, making it difficult to report all cases [33,34]. This is unlike Porto Alegre, where hospital deliveries do not occur in only 0.5% of the occasions [35]. In the present study, it was possible to identify that those regions within the same city with very high development showed statistically lower prevalence of CA compared to the others. Although in medium and high development regions there is greater income diversity in households, which makes this indicator more disperse, very high development regions are characterized by having almost exclusively high-income families [20], which may explain the difference between the observed frequencies. We can assume that women from regions with very high development have greater gestational planning with better preconception and gestational conditions, such as better prenatal coverage, healthy lifestyle habits, greater access to quality food and in sufficient quantity, which reduces the risk of CA considered preventable [3,4,5,36].

Births that occurred in a public hospital and by cesarean section were associated with CA, regardless of local development. This finding may be related to the fact that Porto Alegre has four large public referral hospitals for high-risk pregnancies, which also provide childbirth care for women in situations of greater social vulnerability [20]. Similarly, a study that evaluated national data from Argentina stratifying by public and private hospitals showed that, at the national level, the highest prevalence was observed in public hospitals compared to private ones [28]. Otherwise, many CA are identified during pregnancy, which may be linked to the high number of cesarean sections performed in this population, as has been shown in the literature [1,24,37].

Advanced maternal age poses a risk to the health of the mother and the development of the fetus. In addition to the increased risk of aneuploidy and gestational loss, older women tend to have a higher number of chronic diseases, such as diabetes mellitus and hypertension [7,17,27]. Different studies show that the higher the maternal age, especially above 35 years, the higher the frequency of children with birth defects [5,24,38]. In a case-control study conducted at a referral hospital in Ethiopia, Tsehay et al. [5] identified that women of advanced age were five times more likely to have newborns with some CA compared to those women in the 20–35 age group. In the present study, although the highest proportion of women aged ≥36 years was observed in regions of very high development, in the historical analysis these regions had the lowest prevalence. This shows that, although advanced age is an unavoidable maternal risk factor, it can still be aggravated in women of lower socioeconomic status in less developed regions. In addition, although high proportions of pregnancies with advanced age are observed in the very high development regions in this study, it is known that these pregnancies are mostly planned, with better preconception care and with a considerable prevalence of assisted conceptions, a method that is increasingly common in women with advanced age, mostly performed in the private sector [39]. These findings support the idea that the socioeconomic environment in which the mother is embedded, as well as adequate prenatal care, can reflect on the better development of the fetus [24,28].

No association was observed between recording any CA in adolescent mothers (≤19 years). Several studies show an association of young maternal age with gastroschisis [6,7,40]. In a study conducted in São Paulo, the largest city in Brazil, Cosme et al. [41] identified a lower risk for CA in women at young age (<20 years) compared to those at advanced age (≥40 years). However, in the present study, there was no stratification of CA type, as in the study by Tsehay et al. [5], which indicates that young maternal age represents risk for some specific CA [41].

Low maternal education is consistently cited as a risk factor for complications in pregnancy and lower adherence to recommendations and is usually related to worse socioeconomic conditions [13,14,27,42]. In this study, we observed an association of CA with lower education only in very high development regions (RR 1.27 95% CI 1.03–1.54), suggesting that the mother’s individual choices, such as better adherence to recommendations, may outweigh the exposure of the socioeconomic environment in which she lives. Another hypothesis, which still needs to be investigated, is that these women may represent a small portion of the population who have shown social ascension.

In addition to socioeconomic factors, another aspect that deserves to be highlighted is the important emotional repercussion on children’s families. It is well established that childhood illnesses can increase the risk of psychological distress and mental disorders in parents, especially in the postpartum period, when there are more expectations and adaptations in relation to the newborn. Families with children born with some form of CA require specialized care, and mothers with these demands added to ordinary care present a physical and mental burden [43,44]. This cumulative burden becomes even more concerning when there are pre-existing factors, such as economic challenges, limited family support, and low educational attainment, resulting in little or no access to health services, lack of access to information, and increased uncertainty about their children’s future [43,45].

In the present study, it is also important to highlight some of its strengths. One strength is the fact that it is a time series that used data from a region that is a reference in the quality of SINASC information throughout the country and with a significant “n” [46,47]. In addition, an investigation was conducted in a population of live births in the same city in a time series comparing regions according to the MHDI, whereas most studies evaluate regional and/or socioeconomic differences in the risk of CA through the analyses of different countries, cities, or type of hospital (public and private). However, some limitations were identified. CA were not stratified, but as already observed in previous publications of other population-based epidemiological studies, the total analysis of CA did not interfere with the results and objectives of this study. However, in the future, a more in-depth analysis of the CA found and their relationship with, for example, maternal age, would be opportune. Although there are efforts with protocols and training to improve surveillance of birth defects at birth [9], the number of undetectable CA in the first days of life reflects a weakness in national studies to estimate the frequency of CA, with likely underestimation of values. For this reason, when working with CA, we present prevalence rather than incidence. Another limitation of this study is in relation to the completion of the live births’ declaration related to data that would be useful for the analyses. Although the region presents one of the best completions in the country, it follows a trend towards incompleteness of data, such as ethnicity of the mother and paternal age, which makes it impossible to use these two variables.

The results of this study identified that women from regions with very high development are at lower risk for having children with CA, even though more women are of advanced age. This suggests that although the mother has unavoidable risk factors, an early approach may be a protective factor. Pregnant women, especially those of advanced age, should be encouraged to change their habits, with folic acid supplementation, improved diet, physical activity, smoking cessation, and better control of weight and comorbidities. Although individual maternal exposure factors such as smoking, physical activity, diet, and supplementation were not evaluated, the study draws attention to these recommendations that are more frequently adhered to in women with a higher socioeconomic status.

## 5. Conclusions

Women of advanced age (36 years or more) are at increased risk of having children with CA regardless of maternal schooling and local development. The results indicate that maternal biological characteristics have an important influence on the risk of CA, but social characteristics can also have an influence, as regions with higher development had lower prevalence of CA.

## Figures and Tables

**Table 1:** Distribution of absolute frequency and association of sociodemographic, perinatal, and neonatal characteristics on congenital anomaly according to the MHDI classification in Porto Alegre from 2000 to 2017.

	MHDI											
	MEDIUM				HIGH				VERY HIGH			
	TOTAL *n* (%)	CA *n* (%)	RR[CI95%]	*p*	TOTAL *n* (%)	CA *n* (%)	RR[CI95%]	*p*	TOTAL *n* (%)	CA *n* (%)	RR[CI95%]	*p*
Maternal Age (Years)												
10 to 19	12,003 (21.4)	202 (1.7)	1.22[1.04–1.43]	0.016	34,632 (17.6)	599 (1.7)	1.19[1.09–1.31]	<0.001	8296 (9.1)	136 (1.6)	1.34[1.12–1.61]	0.001
≥36	5399 (9.6)	122 (2.3)	1.64[1.35–1.99]	22,593 (11.5)	<0.001	460 (2.0)	1.40[1.27–1.55]	<0.001	18,971 (20.8)	253 (1.3)	1.09[0.95–1.26]	0.215
20 to 35	38,727 (69.0)	535 (1.4)	1	-	139,765 (70.9)	2024 (1.4)	1	-	64,099 (70.2)	782 (1.2)	1	-
	56,129 (100)	859 (1.5)	-	-	196,990 (100)	3083 (1.6)	-	-	91,366 (100)	1171 (1.3)	-	-
Maternal Education (Years)												
<8	21,378 (38.3)	354 (1.7)	1.27[1.01–1.59]	0.042	62,183 (31.7)	1096 (1.8)	1.48[1.33–1.64]	<0.001	14,145 (15.5)	276 (2.0)	2.08[1.80–2.42]	<0.001
≥8 to 11	27,483 (49.2)	401 (1.5)	1.12[0.89–1.40]	0.336	91,556 (46.6)	1466 (1.6)	1.34[1.21–1.48]	<0.001	27,855 (30.6)	423 (1.5)	1.62[1.42–1.85]	<0.001
≥12	6969 (12.5)	91 (1.3)	1	-	42,548 (21.7)	508 (1.2)	1	-	49,100 (53.9)	460 (0.9)	1	-
	55,830 (100)	846 (1.5)	-	-	196,287 (100)	3070 (1.6)	-	-	91,100 (100)	1159 (1.3)	-	-
Marital Status												
Single/Separated/Widowed	39,733 (71.0)	630 (1.6)	1.15[0.99–1.34]	0.072	131,945 (67.1)	2212 (1.7)	1.26[1.17–1.36]	<0.001	46,605 (51.1)	682 (1.5)	1.35[1.20–1.51]	<0.001
Married/Stable Union	16,230 (29.0)	224 (1.4)	1		64,615 (32.9)	860 (1.3)	1	-	44,576 (48.9)	484 (1.1)	1	-
	55,963 (100)	854 (1.5)	-	-	196,560 (100)	3072 (1.6)	-	-	91,181 (100)	1166 (1.3)	-	-
Parity												
Multipara	34,259 (61.2)	504 (1.5)	0.91[0.79–1.04]	0.161	112,149 (57)	1830 (1.6)	1.10[1.031–1.19]	0.005	43,846 (48.1)	624 (1.4)	1.25[1.12–1.40]	<0.001
Primipara	21,724 (38.8)	352 (1.6)	1	-	84,510 (43.0)	1245 (1.5)	1	-	47,367 (51.9)	539 (1.1)	1	1
	55,983 (100)	856 (1.5)	-	-	196,659 (100)	3075 (1.6)	-	-	91,213 (100)	1163 (1.3)	-	-
Prenatal Care (Number of Consultations)												
None	1955 (3.5)	24 (1.2)	0.84[0.56–1.26]	0.405	6344 (3.2)	128 (2.0)	1.38[1.15–1.64]	<0.001	1875 (2.1)	30 (1.6)	1.38[0.96–1.98]	0.079
1 to 3	5823 (10.4)	101 (1.7)	1.19[0.96–1.47]	0.111	16,917 (8.6)	294 (1.7)	1.19[1.05–1.34]	0.006	4539 (5.0)	85 (1.9)	1.62[1.30–2.02]	<0.001
4 to 6	14,406 (25.8)	239 (1.7)	1.14[0.98–1.33]	0.101	43,538 (22.2)	747 (1.7)	1.17[1.08–1.27]	<0.001	12,947 (14.2)	220 (1.7)	1.47[1.27–1.70]	<0.001
≥7	33,647 (60.3)	491 (1.5)	1	**-**	129,607 (66.0)	1899 (1.5)	1	-	71,807 (78.8)	831 (1.2)	1	**-**
	55,831 (100)	855 (1.5)	-	-	196,406 (100)	3068 (1.6)	-	-	91,168 (100)	1166 (1.3)	-	-
Birth												
Cesarean	20,613 (36.7)	386 (1.9)	1.40[1.23–1.60]	<0.001	86,773 (44.1)	1524 (1.8)	1.24[1.16–1.33]	<0.001	56,237 (61.6)	707 (1.3)	0.95[0.85–1.07]	0.405
Vaginal	35,518 (63.3)	473 (1.3)	1	**-**	110,210 (55.9)	1558 (1.4)	1	**-**	35,127 (38.4)	464 (1.3)	1	-
	56,131 (100)	859 (1.5)	-	-	196,983 (100)	3082 (1.6)			91,364 (100)	1171 (1.3)	-	-
Gestational Age (Weeks)												
<27	276 (0.5)	11 (4.0)	3.01[1.68–5.40]	<0.001	1001 (0.5)	55 (5.5)	3.95[3.05–5.13]	<0.001	420 (0.5)	19 (4.5)	4.03[2.59–6.29]	<0.001
28 to 31	575 (1.0)	19 (3.3)	2.50[1.59–3.91]	<0.001	1897 (1.0)	79 (4.2)	3.00[2.40–3.73]	<0.001	915 (1.0)	33 (3.6)	3.22[2.29–4.52]	<0.001
32 to 36	5122 (9.1)	162 (3.2)	2.39[2.02–2.83]	<0.001	18,022 (9.2)	488 (2.7)	1.95[1.77–2.14]	<0.001	8943 (9.8)	206 (2.3)	2.05[1.77–2.39]	<0.001
≥37	50,027 (89.3)	662 (1.3)	1	-	175,665 (89.3)	2442 (1.4)	1	-	80,936 (88.7)	908 (1.1)	1	-
	56,000 (100)	854 (1.5)	-	-	196,585 (100)	3064 (1.6)	-	-	91,214 (100)	1166 (1.3)	-	-
Apgar (5th Minute)												
<7	746 (1.3)	72 (9.7)	6.79[5.40–8.55]	<0.001	2458 (1.3)	220 (9.0)	6.15[5.39–7.01]	<0.001	765 (0.8)	81 (10.6)	8.85[7.14–10.96]	<0.001
≥7	54,894 (98.7)	780 (1.4)	1	-	193,441 (98.7)	2816 (1.5)	1	-	90,323 (99.2)	1081 (1.2)	1	-
	55,640 (100)	852 (1.5)	-	-	195,899 (100)	3036 (1.5)	-	-	91,088 (100)	1162 (1.3)	-	-
Low Birth Weight												
Yes (<2500 g)	5693 (10.1)	201 (3.5)	2.70[2.32–3.16]	<0.001	19,438 (9.9)	647 (3.3)	2.43[2.23–2.64]	<0.001	8743 (9.6)	278 (3.2)	2.94[2.58–3.36]	<0.001
No (≥2500 g)	50,438 (89.9)	658 (1.3)	1	-	177,554 (90.1)	2436 (1.4)	1	-	82,625 (90.4)	893 (1.1)	1	-
	56,131 (100)	859 (1.5)	-	-	196,992 (100)	3083 (1.6)	-	-	91,368 (100)	1171 (1.3)	-	-
Newborn Sex												
Male	28,892 (51.5)	515 (1.8)	1.45[1.26–1.66]	<0.001	100,771 (51.2)	1747 (1.7)	1.27[1.18–1.36]	<0.001	46,646 (51.1)	665 (1.4)	1.28[1.14–1.44]	<0.001
Female	27,231 (48.5)	336 (1.2)	1	-	96,202 (48.8)	1317 (1.4)	1	-	44,714 (48.9)	498 (1.1)	1	
	56,123 (100)	851 (1.5)	-	-	196,973 (100)	3064 (1.6)	-	-	91,360 (100)	1163 (1.3)	-	-
Type of Hospital												
Public	26,783 (48.1)	499 (1.9)	2.42[1.81–3.23]	<0.001	100,926 (51.7)	1976 (2.0)	2.50[2.22–2.82]	<0.001	30,538 (33.7)	578 (1.9)	2.78[2.43–3.20]	<0.001
Mixed	22,358 (40.2)	293 (1.3)	1.70[1.26–2.29]	<0.001	54,987 (28.1)	718 (1.3)	1.67[1.46–1.90]	<0.001	14,228 (15.7)	225 (1.6)	2.32[1.96–2.76]	<0.001
Private	6487 (11.7)	50 (0.8)	1	-	39,418 (20.2)	309 (0.8)	1	-	45,856 (50.6)	312 (0.7)	1	-
	55,628 (100)	842 (1.5)			195,331 (100)	3003 (1.5)			90,622 (100)	1115 (1.2)	-	-
Pregnancy												
Multiple	1152 (2.1)	25 (2.2)	1.43[0.97–2.12]	0.075	4579 (2.3)	86 (1.9)	1.21[0.98–1.49]	0.084	2859 (3.1)	31 (1.1)	0.84[0.59–1.20]	0.341
Single	54,977 (97.9)	834 (1.5)	1	-	192,406 (97.7)	2997 (1.6)	1	-	88,503 (96.9)	1140 (1.3)	1	-
	56,129 (100)	859 (1.5)	-	-	196,985 (100)	3083 (1.6)	-	-	91,362 (100)	1171 (1.3)	-	-

Dependent variable: congenital anomaly. RR: relative risk. CI95%: 95% confidence interval. MHDI: Municipal Human Development Index. *n*: number of participants. %: percentage. *p*: *p* value. <: less. ≥: greater than or equal to.

**Table 2:** Association of sociodemographic, perinatal, and neonatal characteristics on congenital anomaly according to the MHDI classification in Porto Alegre from 2000 to 2017 (adjusted models).

	Medium MHDI		High MHDI		Very High MHDI	
	RR[CI95%]	*p*	RR[CI95%]	*p*	RR[CI95%]	*p*
Maternal Age (Years)						
10 to 19	1.06[0.88–1.27]	0.552	1.07[0.97–1.20]	0.175	0.99[0.80–1.21]	0.890
≥36	1.60[1.30–1.97]	<0.001	1.40[1.26–1.55]	<0.001	1.28[1.10–1.50]	0.002
20 to 35	1	-	1	-	1	-
Maternal Education (Years)						
<8	1.10[0.85–1.44]	0.460	1.04[0.92–1.18]	0.577	1.27[1.03–1.54]	0.027
≥8 to 11	0.99[0.77–1.27]	0.928	1.00[0.90–1.12]	0.975	1.08[0.91–1.28]	0.349
≥12	1	-	1	-	1	-
Maternal Status						
Single/Separated/Widowed	1.12[0.96–1.32]	0.162	1.17[1.08–1.27]	<0.001	1.01[0.88–1.15]	0.927
Married/Stable Union	1	-	1	-	1	-
Prenatal Care (Number of Consultations)						
None	0.69[0.46–1.06]	0.091	0.93[0.76–1.13]	0.463	0.57[0.38–0.86]	0.008
from 1 to 3	0.88[0.70–1.13]	0.328	0.85[0.74–0.97]	0.018	0.79[0.61–1.01]	0.064
from 4 to 6	0.96[0.82–1.13]	0.617	0.93[0.84–1.01]	0.082	0.82[0.69–0.97]	0.018
≥7	1	-	1	-	1	-
Birth						
Cesarean	1.32[1.14–1.52]	<0.001	1.36[1.26–1.47]	<0.001	1.27[1.12–1.44]	<0.001
Vaginal	1	-	1	-	1	-
Gestational Age (Weeks)						
<27 weeks	0.80[0.40–1.60]	0.520	0.88[0.62–1.25]	0.479	0.77[0.43–1.39]	0.390
28 to 31 weeks	0.86[0.51–1.46]	0.580	1.11[0.85–1.45]	0.440	1.17[0.77–1.79]	0.463
32 to 36 weeks	1.54[1.20–1.98]	0.001	1.29[1.13–1.48]	<0.001	1.29[1.03–1.62]	0.026
≥37	1	-	1	-	1	-
Apgar (5th Minute)						
<7	4.74[3.56–6.30]	<0.001	4.29[3.66–5.04]	<0.001	4.99[3.84–6.51]	<0.001
≥7	1	-	1	-	1	-
Low Weight						
Yes (<2500 g)	1.87[1.46–2.4]	<0.001	1.76[1.54–2.00]	<0.001	2.49[1.99–3.10]	<0.001
No (≥2500 g)	1	-	1	-	1	-
Newborn Sex						
Male	1.43[1.24–1.64]	<0.001	1.29[1.20–1.38]	<0.001	1.28[1.14–1.44]	<0.001
Female	1	-	1	-	1	-
Type of Hospital						
Public	2.48[1.80–3.43]	<0.001	2.70[2.34–3.12]	<0.001	2.94[2.43–3.56]	<0.001
Mixed	1.69[1.20–2.36]	0.002	1.73[1.49–2.02]	<0.001	2.22[1.83–2.70]	<0.001
Private	1	-	1	-	1	-
Pregnancy						
Multiple	0.75[0.49–1.13]	0.171	0.63[0.50–0.80]	<0.001	0.41[0.28–0.61]	<0.001
Single	1	-	1	-	1	-

Dependent variable: congenital anomaly. RR: relative risk. CI95%: 95% confidence interval. MHDI: Municipal Human Development Index. *p*: *p* value <: less. ≥: greater than or equal to. The model was adjusted for maternal age, maternal education, prenatal care, birth, gestational age, Apgar, low weight, newborn sex, type of hospital, and pregnancy.

## Data Availability

The datasets used during and/or analyzed during the current study are not publicly available due to privacy or ethical restrictions but are available from the corresponding author on reasonable request.

## References

[1] World Health Organization (2016). Birth Defects Surveillance Training: Facilitator’s Guide.

[2] Boyle B., Addor M.C., Arriola L., Barisic I., Bianchi F., Csáky-Szunyogh M., de Walle H.E.K., Dias C.M., Draper E., Gatt M. (2018). Estimating Global Burden of Disease due to congenital anomaly: An analysis of European data. Arch. Dis. Child.-Fetal Neonatal Ed..

[3] Lee K.S., Choi Y.J., Cho J., Lee H., Park S.J., Park J.S., Hong Y.C. (2021). Environmental and Genetic Risk Factors of Congenital Anomalies: An Umbrella Review of Systematic Reviews and Meta-Analyses. J. Korean Med. Sci..

[4] Mendes I.C., Jesuino R.S.A., Pinheiro D.D.S., Rebelo A.C.S. (2018). Anomalias congênitas e suas principais causas evitáveis: Uma revisão. Rev. Méd. Minas Gerais.

[5] Tsehay B., Shitie D., Lake A., Abebaw E., Taye A., Essa E. (2019). Determinants and seasonality of major structural birth defects among newborns delivered at primary and referral hospital of East and West Gojjam zones, Northwest Ethiopia 2017–2018: Case-control study. BMC Res. Notes.

[6] Jones A.M., Isenburg J., Salemi J.L., Arnold K.E., Mai C.T., Aggarwal D., Arias W., Carrino G.E., Ferrell E., Folorunso O. (2016). Increasing Prevalence of Gastroschisis–14 States, 1995–2012. Morb. Mortal. Wkly. Rep..

[7] Stallings E.B., Isenburg J.L., Short T.D., Heinke D., Kirby R.S., Romitti P.A., Canfield M.A., O’Leary L.A., Liberman R.F., Forestieri N.E. (2019). Population-based birth defects data in the United States, 2012–2016: A focus on abdominal wall defects. Birth Defects Res..

[8] Moorthie S., Blencowe H., Darlison M.W., Lawn J., Morris J.K., Modell B., Bittles A.H., Christianson A., Cousens S., Gibbons S. (2018). Estimating the birth prevalence and pregnancy outcomes of congenital malformations worldwide. J. Community Genet..

[9] Ministério da Saúde (2021). Boletim Epidemiológico, Anomalias Congênitas no Brasil, 2010 a 2019: Análise de um Grupo Prioritário para a Vigilância ao Nascimento.

[10] Ajao A.E., Adeoye I.A. (2019). Prevalence, risk factors and outcome of congenital anomalies among neonatal admissions in OGBOMOSO, Nigeria. BMC Pediatr..

[11] Ratowiecki J., Santos M.R., Poletta F., Heisecke S., Elias D., Gili J., Gimenez L., Pawluk M., Uranga R., Cosentino V. (2021). Social inequities in teenage mothers and the relationship to adverse perinatal outcomes in South American populations. Cad. Saúde Pública.

[12] Monteiro D.L.M., Monteiro I.P., Machado M.S.C., Bruno Z.V., Silveira F.A.D., Rehme M.F.B., Takiuti A.D., Rodrigues N.C.P. (2021). Trends in teenage pregnancy in Brazil in the last 20 years (2000–2019). Rev. Assoc. Med. Bras..

[13] Koffi A.K., Maina A., Yaroh A.G., Habi O., Bensaïd K., Kalter H.D. (2016). Social determinants of child mortality in Niger: Results from the 2012 National Verbal and Social Autopsy Study. J. Glob. Health.

[14] Mensch B.S., Chuang E.K., Melnikas A.J., Psaki R.S. (2019). Evidence for causal links between education and maternal and child health: Systematic review. Trop. Med. Int. Health.

[15] Geib L.T., Fréu C.M., Brandão M., Nunes M.L. (2010). Social and biological determinants of infant mortality in population cohort in the city of Passo Fundo, Rio Grande do Sul State. Ciência Saúde Coletiva.

[16] França E.B., Lansky S., Rego M.A.S., Malta D.C., França J.S., Teixeira R., Porto D., Almeida M.F., Souza M.F.M., Szwarcwald C.L. (2017). Leading causes of child mortality in Brazil, in 1990 and 2015: Estimates from the Global Burden of Disease study. Rev. Bras. Epidemiol..

[17] Mathews T., MacDorman M.F., Thoma M.E. (2015). Infant mortality statistics from the 2013 period linked birth/infant death data set. Natl. Vital Stat. Rep..

[18] Sitkin N.A., Ozgediz D., Donkor P., Farmer D.L. (2015). Congenital anomalies in low- and middle-income countries: The unborn child of global surgery. World J. Surg..

[19] Adegbosin A.E., Zhou H., Wang S., Stantic B., Sun J. (2019). Systematic review and meta-analysis of the association between dimensions of inequality and a selection of indicators of Reproductive, Maternal, Newborn and Child Health (RMNCH). J. Glob. Health.

[20] Observapoa (2019). Indicadores: Porto Alegre em Análise. http://www.observapoa.com.br/default.php?p_secao=4.

[21] PNUD, IPEA, FJP (2014). Atlas do Desenvolvimento Humano nas Regiões Metropolitanas Brasileiras.

[22] Ministério da Saúde (2009). A Experiência Brasileira em Sistemas de Informação em Saúde.

[23] World Health Organization (2006). Orientation Programme on Adolescent Health for Health Care Providers.

[24] Glick I., Kadish E., Rottenstreich M. (2021). Management of Pregnancy in Women of Advanced Maternal Age: Improving Outcomes for Mother and Baby. Int. J. Women’s Health.

[25] Wit E., Heuvel E.V.D., Romeijn J.W. (2012). ‘All models are wrong…’: An introduction to model uncertainty. Stat. Neerl..

[26] Morris J.K., Springett A.L., Greenlees R., Loane M., Addor M.C., Arriola L., Barisic I., Bergman J.E.H., Csaky-Szunyogh M., Dias C. (2018). Trends in congenital anomalies in Europe from 1980 to 2012. PLoS ONE.

[27] Mai C.T., Isenburg J.L., Canfield M.A., Meyer R.E., Correa A., Alverson C.J., Lupo P.J., Riehle-Colarusso T., Cho S.J., Aggarwal D. (2019). National population-based estimates for major birth defects, 2010–2014. Birth Defects Res..

[28] Bronberg R., Groisman B., Bidondo M.P., Barbero P., Liascovich R. (2021). Birth prevalence of congenital anomalies in Argentina, according to socioeconomic level. J. Community Genet..

[29] Ministério da Saúde (2009). Portaria n° 1.793, de 11 de agosto de 2009. Institui a Comissão Interinstitucional para Implementação, Acompanhamento e Monitoramento das Ações de Fortificação de Farinhas de Trigo, de Milho e de seus Subprodutos. Diário Oficial da União.

[30] Ministério da Saúde (2011). Alguns Documentos Introdutórios Sobre a Rede Cegonha. Distribuição na Oficina Sobre Rede Cegonha no Seminário do CONASEMS.

[31] Raupp L., Adorno R.D.C.F. (2015). Psychotropic territories in the center of Porto Alegre city, Rio Grande do Sul, Brazil. Saúde Soc..

[32] Bastos F.I.B., Bertoni N. (2014). Pesquisa Nacional sobre o uso de crack: Quem são os usuários de crack e/ou similares do Brasil? Quantos são nas capitais brasileiras?. Pesquisa Nacional sobre o Uso de Crack: Quem são os Usuários de Crack e/ou Similares do Brasil? Quantos são nas Capitais Brasileiras?.

[33] Zaganjor I., Sekkarie A., Tsang B.L., Williams J., Razzaghi H., Mulinare J., Sniezek J.E., Cannon M.J., Rosenthal J. (2016). Describing the Prevalence of Neural Tube Defects Worldwide: A Systematic Literature Review. PLoS ONE.

[34] Toobaie A., Yousef Y., Balvardi S., St-Louis E., Baird R., Guadagno E., Poenaru D. (2019). Incidence and prevalence of congenital anomalies in low- and middle-income countries: A systematic review. J. Pediatr. Surg..

[35] Cunha J., Gomes A.L.M., Finkler A., Belló I.L.D.S., Silva K.C.D., Oliveira T.C.M.D. (2017). SINASC Sistema de Informação sobre Nascidos Vivos: Relatório 2015 Geral: Informações Referentes ao Número de Nascidos Vivos em Porto Alegre, Variáveis Maternas, do Parto e do Recém Nascido.

[36] Moraes C.L., Mendonça C.R., Melo N.C.E., Amaral W.N.D. (2019). Prevalence and Association of Congenital Anomalies According to the Maternal Body Mass Index: Cross-Sectional Study. Rev. Bras. Ginecol. Obs..

[37] Vanassi B.M., Parma G.C., Magalhaes V.S., Santos A.C.C.D., Iser B.P.M. (2021). Congenital anomalies in Santa Catarina: Case distribution and trends in 2010–2018. Rev. Paul. Pediatr..

[38] Kishimba R.S., Mpembeni R., Mghamba J. (2015). Factors associated with major structural birth defects among newborns delivered at Muhimbili National Hospital and Municipal Hospitals in Dar Es Salaam, Tanzania 2011–2012. Pan Afr. Med. J..

[39] Machin R., Mendosa D., Augusto M.H.O., Monteleone P.A.A. (2020). Assisted Reproductive Technologies in Brazil: Characterization of centers and profiles from patients treated. JBRA Assist. Reprod..

[40] dos Reis L.V., Araujo Júnior E., Guazzelli C.A., Cernach M.C., Torloni M.R., Moron A.F. (2015). Congenital Anomalies Detected at Birth in Newborns of Adolescent Women. Acta Med. Port..

[41] Cosme H.W., Lima L.S., Barbosa L.G. (2017). Prevalence of congenital anomalies and their associated factors in newborns in the city of São Paulo from 2010 to 2014. Rev. Paul. Pediatr..

[42] Anele C.R., Hirakata V.N., Goldani M.Z., da Silva C.H. (2021). The influence of the municipal human development index and maternal education on infant mortality: An investigation in a retrospective cohort study in the extreme south of Brazil. BMC Public Health.

[43] Kolaitis G.A., Meentken M.G., Utens E.M.W.J. (2017). Mental Health Problems in Parents of Children with Congenital Heart Disease. Front. Pediatr..

[44] Kasparian N.A., Kan J.M., Sood E., Wray J., Pincus H.A., Newburger J.W. (2019). Mental health care for parents of babies with congenital heart disease during intensive care unit admission: Systematic review and statement of best practice. Early Hum. Dev..

[45] Benny C., Yamamoto S., McDonald S., Chari R., Pabayo R. (2022). Modelling Maternal Depression: An Agent-Based Model to Examine the Complex Relationship between Relative Income and Depression. Int. J. Environ. Res. Public Health.

[46] Paes N.A., Santos C.S.A.D. (2010). Birth statistics and maternal and infant risk factors in the micro-regions of Northeast Brazil: A factor analysis study. Cad. Saúde Pública.

[47] Pedraza D.F. (2012). Quality of the Information System on Live Births /SINASC: A critical analysis of published studies. Ciênc. Saúde Coletiva.

